# Analysis of HER2 genomic binding in breast cancer cells identifies a global role in direct gene regulation

**DOI:** 10.1371/journal.pone.0225180

**Published:** 2019-11-20

**Authors:** Aisling M. Redmond, Soleilmane Omarjee, Igor Chernukhin, Muriel Le Romancer, Jason S. Carroll

**Affiliations:** 1 University of Cambridge, Cancer Research UK Cambridge Institute, Cambridge, United Kingdom; 2 Université Lyon 1, Lyon, France; 3 Inserm U1052, Centre de Recherche en Cancérologie de Lyon, Lyon, France; 4 CNRS UMR5286, Centre de Recherche en Cancérologie de Lyon, Lyon, France; Università degli Studi di Milano, ITALY

## Abstract

HER2 is a transmembrane receptor tyrosine kinase, which plays a key role in breast cancer due to a common genomic amplification. It is used as a marker to stratify patients in the clinic and is targeted by a number of drugs including Trastuzumab and Lapatinib. HER2 has previously been shown to translocate to the nucleus. In this study, we have explored the properties of nuclear HER2 by analysing the binding of this protein to the chromatin in two breast cancer cell lines. We find genome-wide re-programming of HER2 binding after treatment with the growth factor EGF and have identified a *de novo* motif at HER2 binding sites. Over 2,000 HER2 binding sites are found in both breast cancer cell lines after EGF treatment, and according to pathway analysis, these binding sites were enriched near genes involved in protein kinase activity and signal transduction. HER2 was shown to co-localise at a small subset of regions demarcated by H3K4me1, a hallmark of functional enhancer elements and HER2/H3K4me1 co-bound regions were enriched near EGF regulated genes providing evidence for their functional role as regulatory elements. A chromatin bound role for HER2 was verified by independent methods, including Proximity Ligation Assay (PLA), which confirmed a close association between HER2 and H3K4me1. Mass spectrometry analysis of the chromatin bound HER2 complex identified EGFR and STAT3 as interacting partners in the nucleus. These findings reveal a global role for HER2 as a chromatin-associated factor that binds to enhancer elements to elicit direct gene expression events in breast cancer cells.

## Introduction

Human epidermal growth factor receptor 2 (HER2) is a member of the epidermal growth factor (EGF) family of receptor tyrosine kinases (ErbBs), which traditionally has been known as a transmembrane tyrosine kinase receptor involved in signalling to the mitogen activated protein kinase (MAPK) pathway and the phosphatidylinositol 3-kinase (PI3K) pathway. HER2 has no known ligand but heterodimerises with other ErbB receptors when they are activated by ligand. HER2 is amplified in a number of breast cancer tumours, with the frequency reported to range from 10% [1] to 30% [2]. Breast cancer patients with this amplification of HER2 have a significantly poorer prognosis when compared to patients with non-amplified HER2 [2]. As a result of its amplification and the location of HER2 in the extracellular membrane, a number of therapies have been developed to target this molecule. The monoclonal antibody trastuzumab has been used in the clinic to treat patients with HER2-positive breast cancer for a number of years [3]. More recently, the small molecule tyrosine kinase inhibitor lapatinib, which targets both HER2 and EGFR, and trastuzumab emtansine (TDM1), an antibody-drug conjugate, have been introduced as a therapy for patients with metastatic breast cancer [4, 5].

The presence of HER2 in the nucleus was first noted by Xie and Hung [6], who also reported that HER2 can function as a transcriptional activator. Giri *et al* [7] reported that HER2 is transported to the nucleus by endocytosis, involving importin β1 and the nuclear pore protein Nup358. Once in the nucleus, HER2 acts as a coactivator for signal transducer and activator of transcription 3 (STAT3), in a complex alongside the progesterone receptor (PR) and AP-1 [8–10]. These interactions were analysed via chromatin immunoprecipitation (ChIP) at the *cyclin D1* promoter in the mouse mammary C4HD cell line [8, 9] and at the *p21CIP1* promoter in the T47D human breast cancer cell line [10]. HER2 has also been reported to bind to the *COX-2* promoter in the breast cancer cell lines BT474 and SKBR3, and the ovarian cancer cell line SKOV3 [11]. In metastatic breast cancer cell lines, Venturutti *et al* [12] demonstrated that HER2 binds to the promoter region of a microRNA, in conjunction with STAT3. In addition, Li *et al* [13] have reported that HER2 associates with β-actin and RNA pol I, resulting in enhanced rRNA gene transcription. In the nucleus of a number of cancer cell lines, HER2 has been found to interact with macrohistone 2A1.2 [14]. Despite the fact that a number of these publications show that the proportion of HER2 in the nucleus appears to be quite low in comparison to that at the membrane, there appears to be an important role for HER2 in the nucleus.

Further studies in clinical samples revealed the presence of nuclear HER2 (nHER2) in tumours from breast cancer patients [15, 16]. Schillaci *et al* [16] reported nHER2 in 33.6% of primary invasive breast carcinomas and this expression was a significant independent predictor of worse overall survival in patients with membranous HER2 (mHER2). Dillon *et al* [15] demonstrated an association between COX-2 expression and nHER2 in a large breast cancer patient population, and nHER2 predicted poor disease-free survival in patients on endocrine treatment. The role of nHER2 has recently been reported to be involved in resistance to trastuzumab, with nHER2 forming a complex at the DNA with STAT3 and ErbB3 [17].

These previous reports in cell lines and breast cancer samples indicate a role for nHER2 in disease progression. However, the HER2-chromatin interactions have focused mainly on binding at specific loci of interest. We mapped nHER2 on a genome-wide scale for the first time, using ChIP-exonuclease (ChIP-exo) [18], a high resolution transcription factor mapping protocol. We chose breast cancer cell lines with amplification of the *ErbB2* locus which represent different breast cancer subtypes, in order to analyse the importance of nHER2 in both of these molecular environments. Using this approach, we sought to identify direct transcription target genes of this important and complex factor.

## Results

### Global binding analysis of HER2 in SKBR3 cells reveals a de novo binding motif

**Fig 1:**
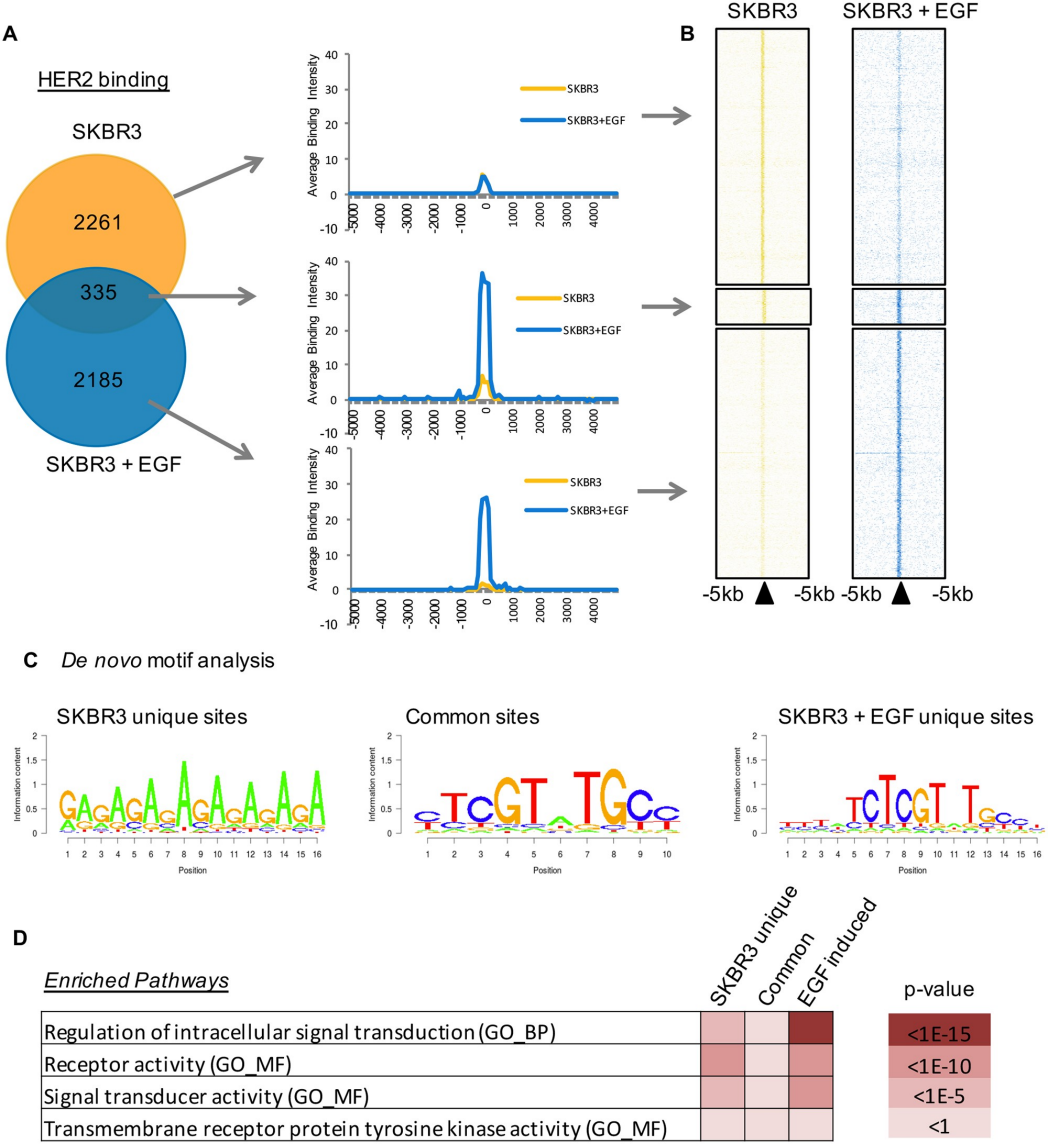
ChIP-exo reveals HER2 binding across the genome of the SKBR3 breast cancer cell line. Experiments were performed in asynchronous conditions, with the supplementation of vehicle (H2O) or EGF (100ng/ul) for 3 hours. A) Venn diagram illustrating the overlap of peaks found in the vehicle treated and EGF treated conditions and average intensity plots generated from the peaks within each subset. EGF treatment re-programmes binding to new sites and increases the average intensity of binding. B) Heatmap illustrates the binding in the different subsets of peaks. C) *De novo* motif analysis of the underlying DNA at the HER2 binding sites. A novel part-palindromic motif (CGT-TGC) was identified at the common binding sites and those found after EGF treatment. D) Gene Ontology analysis of the genes found within 10kb of the binding sites were analysed using the Broad Institute’s GSEA website. Signal transduction and receptor activity pathways feature in particular near the binding sites identified in EGF treated cells SKBR3 cells.

The association of the full-length transmembrane receptor HER2 with the DNA in breast cancer cells was first confirmed by chromatin fractionation and Western blotting. The cell lines SKBR3 and BT474 which have an amplified *ErbB2* locus were used in this project. In these two breast cancer cell lines, HER2 was bound to the chromatin in both vehicle and EGF stimulated conditions (S1A Fig), confirming previous findings showing that full-length HER2 can be chromatin bound [6, 8–10]. Interestingly, ChIP-seq of HER2 in SKBR3 cells was not sensitive enough to identify HER2 binding sites (S1B Fig) but the improved signal from ChIP-exo [18] permitted global mapping. Cells were treated with EGF for three hours (S1C Fig) and HER2 ChIP-exo was conducted (Fig 1, S1D Fig), with peaks called using MACS [19]. In vehicle-treated (asynchronous) and EGF-treated cells, 2,596 and 2,520 binding sites were identified respectively but with only a small overlap (~15%) (Fig 1A and 1B). EGF treatment for 3 hours induced binding at a number of new sites in the genome. In addition, the average binding intensity increased in the presence of this growth factor, evident in the heatmap and in the corresponding average intensity plots (Fig 1A and 1B).

*De novo* motif analysis revealed a motif underlying the binding sites found in the EGF condition and in the common sites bound in both treatment conditions (Fig 1C). This binding site has no known corresponding transcription factor. The motif appears to be palindromic in the core ’CGT_TGC’ sequence. Notably, this motif was not found in the SKBR3 asynchonous only subset of binding sites. This finding, along with the low intensity of these binding sites could indicate a lesser functional role of these binding sites. Analysis of the genes within 10 kb of the binding sites revealed significant associations with genes in pathways involving signal transduction and receptor/kinase activity (Fig 1D). Binding of HER2 was not found to be enriched at any particular genomic feature such as promoters in comparison to the breakdown of the whole genome into these features (S1E Fig).

### EGF treatment induces increased binding in the BT474 breast cancer cell line, with a considerable overlap with those found in the SKBR3 cell line

**Fig 2:**
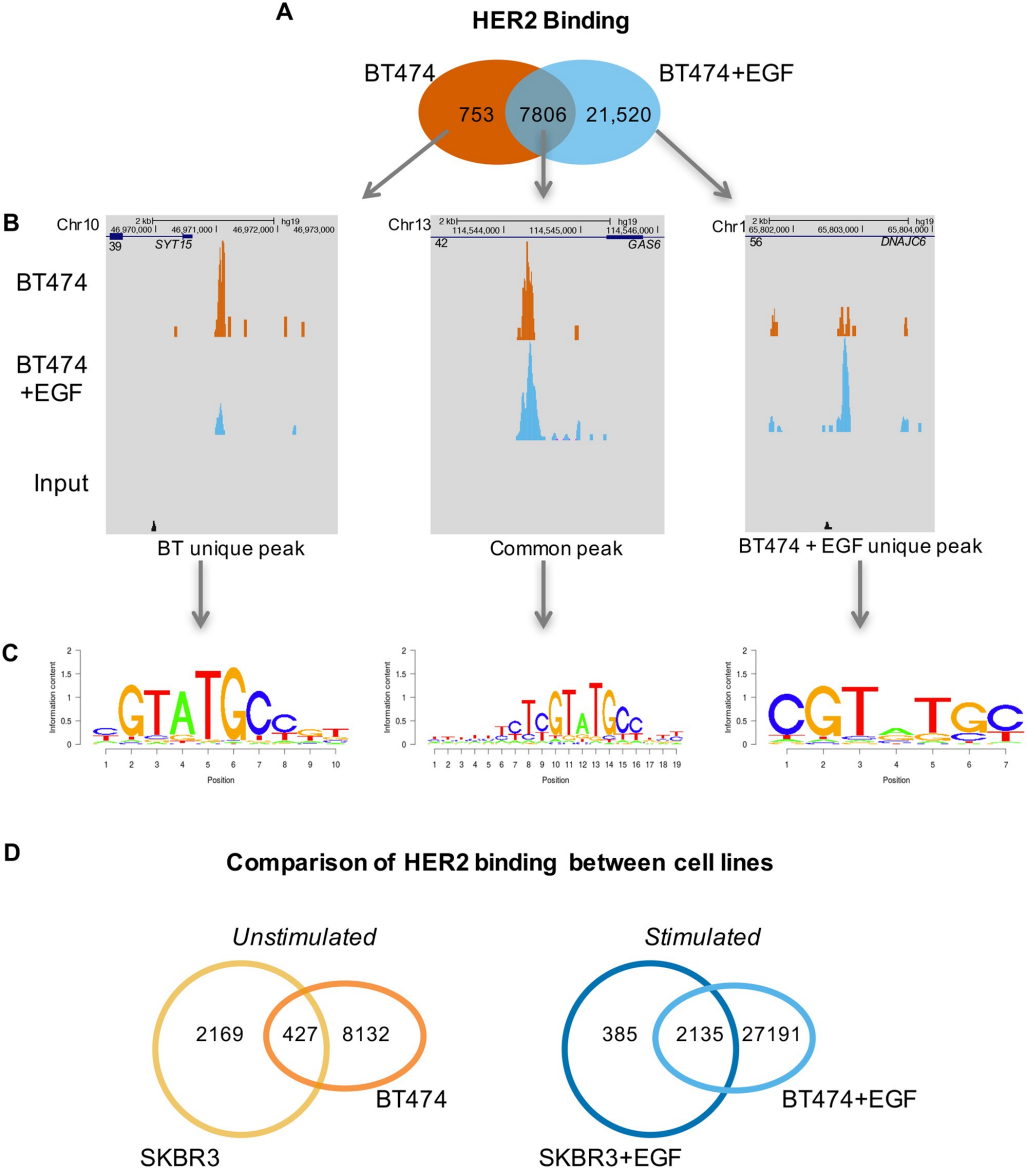
Binding analysis of HER2 in the BT474 cell line. Binding analysis of HER2 in the BT474 cell line reveals a considerable increase in binding after EGF treatment, and shared a number of sites with the SKBR3 cell line. ChIPexo experiments were performed in asynchronous conditions, with the supplementation of vehicle (H2O) or EGF (100ng/ul) for 3 hours. A) Venn diagram illustrating the overlap between the two treatment conditions in the BT474 cell line. EGF treatment induces a considerable increase in binding sites. B) UCSC genome browser shots of unique and common sites for all three subsets of peaks. C) *De novo* motif analysis reveals the same novel motif found in the SKBR3 cells underlying the binding sites in the BT474 cell line. D) Venn diagrams comparing the binding sites found in the two breast cancer cell lines, SKBR3 and BT474, under asynchronous conditions (left) and EGF treatment conditions (right). In asynchronous cells, the majority of the sites are independent, while after EGF treatment, the majority of SKBR3 binding sites are also found in the BT474 cells.

HER2 ChIP-exo was examined in another breast cancer cell line with an amplified *ErbB2* locus, the BT474 cells. This cell line also expresses the progesterone receptor which has previously been reported to associate with HER2 at the chromatin [8–10]. HER2 peaks were evident in both asynchronous and EGF-treated conditions (Fig 2A and 2B). The number of binding sites of HER2 in the BT474 cell line increased considerably with treatment of EGF, however the common sites found in both conditions had the strongest average intensity (Fig 2A & S2A Fig). *De novo* motif analysis of the HER2 binding peaks revealed that all three subsets of binding peaks had a similar underlying motif to that found in the SKBR3 cells, implying a common mechanism for HER2 chromatin binding (Fig 2C). Binding of HER2 in this cell line did not show any enrichment to specific regions of the genome (S2B Fig). Interestingly, upon comparison of the binding in the two breast cancer cell lines under un-treated conditions, (Fig 2D, left) the majority of the peaks were unique to each cell line. In contrast, after treatment with EGF (Fig 2D, right) the majority (85%) of the SKBR3 peaks were also found in the BT474 cell line.

### A subset of HER2 binding sites overlap with enhancer regions and are found near genes involved in signalling pathways

**Fig 3:**
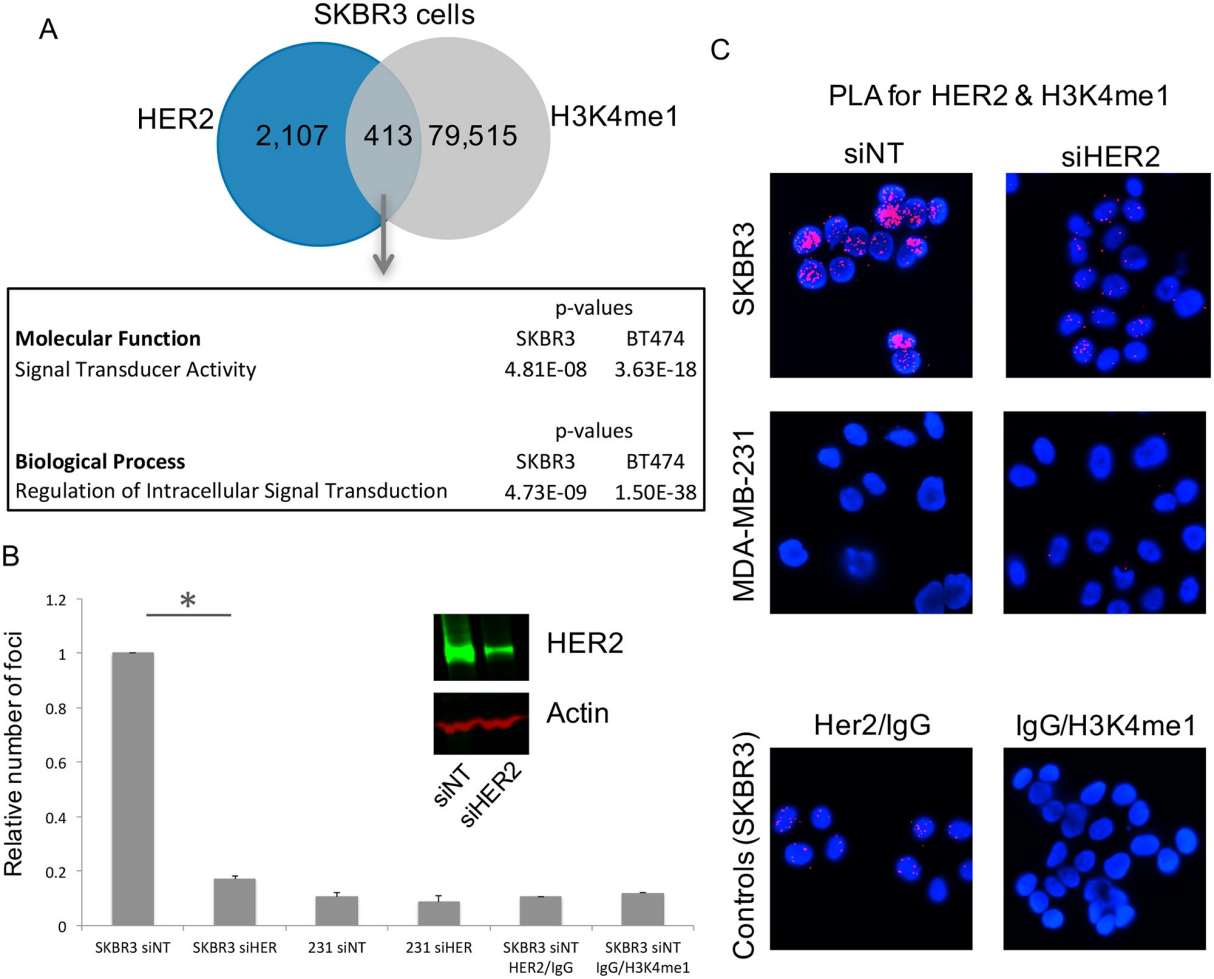
HER2 and H3K4me1 co-localise at the chromatin. HER2 and H3K4me1 co-localise at the chromatin near genes involved in signal transduction and protein kinase activity. A) Venn diagram illustrating the overlapping sites of HER2 and H3K4me1 in the SKBR3 cells. Genes with common binding sites within 10kb of the TSS were analysed using the Broad Institute’s GSEA website, revealing significant enrichment for Signal Transduction GO pathways. B) Proximity ligation assay utilising antibodies raised against HER2 and H3K4me1 illustrating a reduction in the number of fluorescent foci with knockdown using siRNA against HER2. Anti-HER2 (mouse monoclonal, Abcam ab16901) and anti-H3K4me1 (rabbit polyclonal, Abcam ab8895) antibodies were used for PLA experiments. Histogram with quantification of fluorescent foci. *p-value < 0.05 (Student’s t-test). Inset: confirmation of knockdown of HER2. C) Examples of fluorescent images generated from PLA. HER2-negative MDA-MB-231 cells were used as negative control cell line. Additional controls included substituting the individual primary antibodies with IgG (bottom panel).

Monomethylation of lysine 4 on histone 3 (H3K4me1) is enriched at enhancer regions in the genome [20]. Given the fact that HER2 binding occurred throughout the genome, we hypothesised that HER2 binding might occur at enhancers. H3K4me1 was mapped in EGF-treated SKBR3 and BT474 breast cancer cell lines. Two replicates were performed for each cell line. Comparison of the binding of this epigenetic mark with HER2 binding sites, revealed 413 common regions in the SKBR3 cell line, representing 16.4% of the HER2 peaks (Fig 3A). In the BT474 cell line, 3,976 regions were co-occupied by HER2 and H3K4me1, representing 13.6% of all the HER2 bound regions in this specific cell line (S2C Fig). As such, a small proportion of the HER2 bound regions are bona fide enhancer elements.

Genome-wide mapping of HER2 revealed thousands of regions of the genome occupied by this receptor in two independent breast cancer cell lines. As previously shown (S1A Fig) chromatin fractionation followed by Western blotting confirmed HER2 chromatin occupancy. We explored an additional independent method for assessing HER2-chromatin interactions, specifically proximity ligation assay (PLA). PLA permits sensitive identification of *in situ* protein-protein interactions based on physical proximity [21]. We established a PLA assay to assess interactions between HER2 and histone H3K4me1. In the first instance, we confirmed that EGF treatment increased the interaction of HER2 and H3K4me1 in comparison to control cells (S2D Fig). Subsequently, we performed PLA in EGF-stimulated SKBR3 cell lines using HER2 siRNA, which was effective at silencing the HER2 protein (Fig 3B). Interactions between HER2 and H3K4me1 were observed using PLA and silencing of HER2 significantly reduced the number of immunofluorescent foci observed in the nuclei of the EGF-treated breast cancer cells (Fig 3B and 3C). MDA-MB-231, a cell line which does not express HER2, was used as a negative control and did not show any signal from PLA experiments conducted between HER2 and H3K4me1.

Genes within 10kb of the common HER2 and H3K4me1 binding sites revealed 222 genes in SKBR3 cells and 2,123 genes in the BT474 cell line. Gene Set Enrichment Analysis [22, 23] showed that both of these gene lists were enriched for Gene Ontology terms involving signal transduction (Fig 3A). Included in the lists of genes were important signalling proteins such as *MAP3K13*, *PRKCH*, *Myc and MAPK11*.

### Proteomic analysis of HER2 binding partners at the chromatin reveals interactions with STAT3 and EGFR

**Fig 4:**
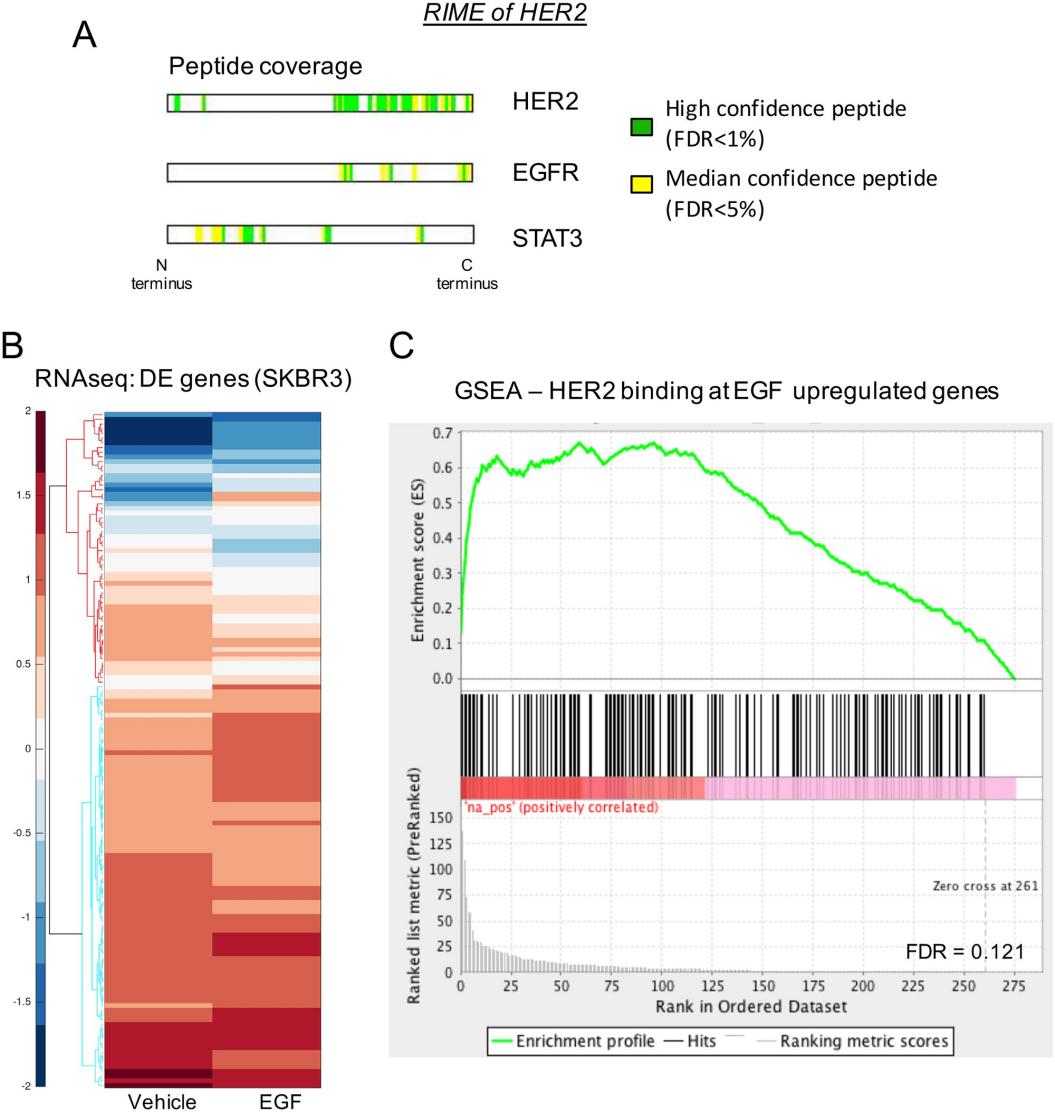
Mass spectrometry and RNAseq reveal STAT3 and EGFR as interacting partners at the chromatin. Mass spectrometry and RNAseq reveal STAT3 and EGFR as interacting partners at the chromatin, with a subsequent influence on expression of nearby genes. A) Peptide coverage of HER2, EGFR, STAT3 from the RIME data from SKBR3 cells. Green and yellow bars represent regions of the full-length protein sequence in which the peptides were identified. Green indicates a high confidence (FDR<1%) peptide and yellow indicates a median confidence peptide (FDR<5%). B) Heatmap from RNAseq in the SKBR3 cell line of the differentially expressed genes which have a HER2 and H3K4me1 overlapping binding site either within the gene body or within 10kb upstream from the TSS. Fifty-nine genes were upregulated and 314 genes were downregulated. C) GSEA analysis combining genes upregulated upon EGF treatment from RNAseq data with the overlapping HER2/H3K4me1 binding sites. A correlation between the ChIP peaks and the upregulated genes in RNAseq is illustrated. Normalised Enrichment Score was 1.0976515.

Having established the binding of HER2 to chromatin in two breast cancer cell lines, a modified rapid immunoprecipitation mass spectrometry of endogenous proteins (RIME) protocol was used to identify partners of HER2 at the chromatin [24]. Two replicates were performed, each using a different nuclear/chromatin preparation kit and the cells were labelled using stable isotope labelling by amino acids in cell culture (SILAC). Asynchronous SKBR3 cells were grown in SILAC medium and treated with vehicle or EGF for 3 hours. Following HER2 purification combined with MS analysis, HER2 was revealed as the top protein, validating the purification approach and the antibody used for ChIP-exo. Peptide coverage of the HER2 protein is shown in Fig 4A. EGFR, a known interactor of HER2 at the membrane was also found to interact with HER2 at the chromatin (Table 1, S1 Table). Importantly, the transcription factor, STAT3, was confirmed to interact with HER2, suggesting potential global functional interplay between these two proteins, in support of the locus specific evidence linking these factors [8]. The interaction of HER2 with EGFR and STAT3 in the nucleus was confirmed by coimmunoprecipitation in the SKBR3 cell line (S2D Fig).

**Table 1:** Mass spectrometry results from SKBR3 cells detailing the number of unique peptides detected for HER2, STAT3 and EGFR.

	JP602/HER2 IP	JP603/IgG	JP628/HER2 IP	JP629/IgG
HER2	32	1	28	0
STAT3	6	1	10	0
EGFR	4	0	5	0

### Genomic binding of HER2 and H3K4me1 correlates with expression of nearby genes

In order to test whether the HER2 genomic binding events had a functional impact, HER2 binding was coupled with gene expression in the SKBR3 cell line. The SKBR3 breast cancer cell line was treated with vehicle or EGF for 3 hours and total RNA was collected for RNA-seq. Five replicates were conducted. The comparison of these samples allowed us to determine which genes were regulated by EGF expression. A total of 7,271 genes were differentially regulated in SKBR3 cells by EGF treatment (FDR 0.01). In order to look at the effect of the HER2 and H3K4me1 binding on these differentially regulated genes, we used two different approaches. Specifically, we focused on genes that had both HER2 binding and H3K4me1 signal within the gene body or up to 10kb upstream from the transcription start site, resulting in a total of 373 genes. A number of these genes were differentially expressed (59 upregulated, 314 downregulated) upon EGF treatment of the SKBR3 cell line, as illustrated by heatmap (Fig 4B). Gene set enrichment analysis revealed that HER2- and H3K4me1-occupied genes were also the genes that were upregulated by EGF stimulation (Fig 4C), suggesting that the HER2 binding occurred at functional enhancer elements, as demarcated by H3K4me1 and that these regions become transcriptional regulatory elements.

## Discussion

The membrane receptor HER2 is a standard clinicopathological variable that is used to stratify breast cancer patients onto specific treatment regimes. While the presence of this molecule in the nucleus of cells has been noted in a handful of publications, this is the first time that global analysis of HER2 binding has been reported. Analysis of the binding of HER2 was achieved using the ChIP-exo method, which increases the sensitivity of ChIP based mapping approaches. In this study we have profiled HER2 binding to the chromatin in two breast cancer cell lines with an amplified *ErbB2* locus. In the SKBR3 cell line, binding was observed in both asynchronous cells and following treatment with EGF. The majority of sites in EGF conditions were new binding sites for HER2, indicating a re-programming of the binding in the presence of EGF supplementation. In contrast, in the BT474 cell line, although EGF induced a large number of new binding sites for HER2, the majority of the binding sites found in asynchronous cells were also found in EGF-treated cells. Notably, the new sites induced by EGF treatment did not have a high average intensity. Importantly, *de novo* motif analysis revealed a novel motif underlying the peaks in both cell lines. Wang et al [11] reported a HER2-associated sequence (HAS) of ‘TCAAATTTC’ in the promoter of the *COX2* gene. This differs considerably to the motif which we have identified in this study. However, we have analysed the sequence which is bound by HER2 across hundreds of sites and in two different cell lines.

As EGF does not act as a direct ligand for HER2, this result could indicate that binding of HER2 to the chromatin is linked to dimerization with EGFR. EGFR itself has previously been reported to also localise to the nucleus in human squamous carcinoma and breast cancer cells [25]. In support of this hypothesis, our proteomic purification analysis revealed an interaction between HER2 and EGFR on the chromatin. Previous reports have identified a shorter variant of ErbB3 interacting with the Cyclin D1 promoter region, and the full length ErbB3 interacting with HER2 in the nucleus [17, 26]. In our study, we did not find an interaction between ErbB3 and HER2 in our mass spectrometry results but this may be due to stimulation with EGF rather than Heregulin, the ligand for ErbB3. The majority of the HER2 binding sites found in the EGF-treated SKBR3 cell line were also found in the BT474 cell line. There were over two thousand common peaks found in both cell lines and these shared binding regions had the highest signal intensity when compared to cell line specific HER2 binding sites, suggesting that the co-occurring HER2 binding sites are the strongest chromatin binding sites and likely play the most important functional role.

In order to gain confidence that the HER2 binding regions were functionally important, we correlated our genome-wide binding information with H3K4me1 ChIP-seq data, since H3K4me1 demarcates functional enhancer elements [20]. We found that approximately 16.4% of the EGF induced HER2 peaks in SKBR3 cells overlapped with H3K4me1. Interestingly, these common peaks of HER2 and H3K4me1 were found to be enhanced near genes classified in ‘Signal Transduction’ functions, suggesting that HER2 might directly regulate transcription of gene expression programmes that represent the same kinase signalling pathways that are potentially activated by the membrane HER2 pathway.

Previous studies have reported HER2 and EGFR translocation to the nucleus involving the integral trafficking from the endoplasmic reticulum to the nuclear envelope transport (INTERNET) pathway [7, 27]. This involves internalisation of the transmembrane receptor from the cell surface by endocytosis, movement from the Golgi to the endoplasmic reticulum and translocation to the inner nuclear membrane. A number of proteins have been reported to be involved in this process, including Importin β1, Nup358 and Sec61 [7, 27]. There is the possibility that the INTERNET process is involved in translocating both HER2 and EGFR as a heterodimer to the nucleus. Interaction with HER2 and the transcription factor STAT3 was also observed in our unbiased proteomic purification analysis and STAT3 has previously been reported to localise with HER2 at the chromatin and may be a critical functional partner of the complex, playing a direct role in gene regulation [8]. How chromatin bound HER2 contributes to this gene regulation is unknown but it could possibly involve phosphorylation and subsequent activation of partner proteins or histones. Members of the MAPK and JAK/STAT pathways including JAK2 and JNK have previously been reported to phosphorylate histones [28, 29].

Our work has complimented that of others in exploring the role of HER2 at the chromatin, confirming that apart from the important canonical role of HER2 in signal transduction at the membrane, there is another aspect to this receptor tyrosine kinase.

## Conclusion

This study provides the first genome-wide binding analysis of the transmembrane receptor HER2. We discovered over two thousand HER2 binding events that were common in two independent breast cancer cell lines, and found colocalisation with the enhancer mark H3K4me1. In addition, HER2 binding correlated with gene expression events induced by EGF. Our understanding of the role of HER2 in breast cancer has mainly focused on the classic view of HER2 as a transmembrane receptor integrated in the plasma membrane of the cell. Our work and that of others have demonstrated that this simplified model is incomplete and ignores the action of HER2 within the nucleus and on the chromatin. Further investigation is required to determine the exact mechanism of HER2 activity within the nucleus.

## Materials and methods

### Cell lines and culturing

SKBR3 and BT474 human cell lines were obtained directly from American Type Culture Collection. SKBR3 cells were grown in McCoys 5A Glutamax, supplemented with 10% FBS. BT474 cells were grown in RPMI-1640, supplemented with 10% FBS. All cell lines were regularly genotyped using STR profiling using the Promega GenePrint 10 system. Cell lines were regularly tested for mycoplasma infection. Cells were treated, without any serum deprivation, for 3 hours with vehicle (H2O) or EGF (100ng/ml, Sigma-Aldrich, St Louis, MO, USA).

### ChIP-exo and ChIP-seq

ChIP-exo and ChIP-seq experiments were performed as previously described [18, 30]. In brief, for ChIP-exo, formaldehyde cross-linked cells were lysed with a series of buffers to isolate the chromatin which was then sonicated. The chromatin was added to antibody-bead complexes and rotated at 4°C overnight. Beads with antibody/protein/chomatin bound were washed in RIPA buffer (50mM HEPES pH 7.6, 1mM EDTA, 0.7% Na deoxycholate, 1% NP-40, 0.5M LiCL) six times, then twice in 10mM Tris HCl pH8.0. End repair, P7 adapter ligation and nick repair were performed on the beads, followed by the Lambda and RecJf exonuclease reactions. Chromatin bound to the antibody of choice was eluted/reverse crosslinked at 65°C overnight. A phenol:chloroform extraction was performed followed by second strand synthesis, P5 adapter ligation and PCR amplification. DNA of 200-350bp was selected and extracted from an agarose gel. Antibodies used were anti-HER2 (Santa Cruz, sc-284, lots numbers F0512, I2712, K0711, B1413) and anti-H3K4me1 (Abcam, ab8895, lot number GR149140-1). For each ChIP-exo/seq experiment, 10 μg (HER2) or 5 μg (H3K4me1) of antibody was used.

### ChIP-exo and ChIP-seq data analysis

Single-end 36 bp reads were generated on the Illumina Hi-Seq2500 and mapped to hg19 genome using bowtie2 2.2.6 [31]. Aligned reads with the mapping quality less than 5 were filtered out. Peaks were called with MACS2 version 2.0.10.20131216 [19] for HER2 data and Sicer version 1.1 [32] for the histone mark data. using sequences from the SKBR3 and BT474 chromatin extracts as a background input control. A further filtration was carried out by removing reads falling into the ‘blacklist’ regions identified by ENCODE [33] or in regions found to have high signals in cell-line-specific input samples as determined by the Bioconductor package *GreyListChIP* (http://www.bioconductor.org/packages/release/bioc/html/GreyListChIP.html). The peaks yielded with MACS2 q value < = 1e-3 were selected for downstream analysis. Meme version 4.9.1 [34] was used to detect known and discover novel binding motifs amongst tag-enriched sequences. The Broad Institute’s Gene Set Enrichment Analysis (GSEA) website (http://software.broadinstitute.org/gsea) was used to perform Gene Ontology studies.

### Rapid IP-mass spectrometry of endogenous protein (RIME)

RIME experiments [24] were performed using two nuclear/chromatin extraction kits. Nuclei Pure Prep Nuclei Isolation kit (Sigma, St Louis, MO, USA) and the chromatin fraction from the Subcellular Extraction kit (Thermo Scientific, Rockford, IL, USA) were used for immunoprecipitating the protein complexes. SKBR3 cells were grown in R/K-deficient SILAC DMEM (PAA; E15-086), 10% dialysed serum (Sigma-Aldrich; F0392), and supplemented with 800mM _L_-lysine ^13^C_6_^15^N_2_ hydrochloride and 482mM _L_ -arginine ^13^C_6_^15^N_4_ hydrochloride (Sigma- Aldrich) for ‘heavy’-labelled media or 800mM _L_-lysine ^12^C_6_^14^N_2_ hydrochloride and 482mM _L_-arginine ^12^C_6_^14^N_4_ hydrochloride for ‘light’-labelled media. Antibody used was anti-HER2 (sc-284, Santa Cruz, lot number B1413). Each RIME experiment was performed by mixing cells from each label after respective drug treatments. One replicate was performed for each extraction kit, with the SILAC labels switched between the treatments. Bead-bound proteins were digested by the addition of 10 ml trypsin solution 15 ng /ul (Worthington Biochemicals) in 100mM ammonium bicarbonate. The beads were then incubated at 37°C overnight. A second step digestion was performed the following day for 4 h. Sample tubes were placed on a magnetic rack and the supernatant solution was collected and acidified by the addition of 2 ml 5% formic acid. The samples were then cleaned using Ultra-Micro C18 Spin Columns (Harvard Apparatus) prior to the mass spectrometry (MS) analysis according to manufacturer’s instructions.

MS was performed using an LTQ Velos-Orbitrap MS (Thermo Scientific) coupled to an Ultimate RSLCnano-LC system (Dionex). Optimal separation conditions resulting in maximal peptide coverage were achieved using an Acclaim PepMap 100 column (C18, 3 μm, 100 Å) (Dionex) with an internal diameter of 75 μm and capillary length of 25 cm. A flow rate of 350 nl/min was used with a solvent gradient of 5% B to 50% B in 120 min. Solvent A was 0.1% (v/v) formic acid in water, whereas the composition of solvent B was 80% (v/v) acetonitrile in 0.1% (v/v) formic acid.

The mass spectrometer was operated in positive ion mode using an Nth order double-play method to automatically switch between Orbitrap-MS and LTQ Velos-MS/MS acquisition. Survey full-scan MS spectra (from 400 to 1,600 m/z) were acquired in the Orbitrap with resolution (R) 60,000 at 400 m/z. The method allowed sequential isolation of the 10 most intense ions for fragmentation in the linear ion trap, depending on signal intensity, using CID at collision energy 30. Target ions already selected for MS/MS were dynamically excluded for 20 s. General MS conditions were electrospray voltage, 2.0kV with no sheath or auxiliary gas flow, an ion selection threshold of 1,000 counts for MS/MS, an activation Q value of 0.25, activation time of 10 ms and an S-Lens RF level of 65% were also applied.

### RIME analysis

Raw MS data files were processed using Proteome Discoverer v.1.4 (Thermo Scientific). Processed files were searched against the SwissProt human database using the Sequest HT. The Nodes for SequestHT included the following parameters: Precursor Mass Tolerance 20ppm, Fragment Mass Tolerance 0.5 Da, Dynamic Modifications were Oxidation of M (+15.995 Da) and Deamidation of N, Q (+0.984 Da). Protein quantification was based on the relative precursor intensity of the light and heavy R(10), K(8) SILAC labelled versions of their matching peptides. The level of confidence for peptide identifications was estimated using the Percolator node with decoy database search. FDR<1% was applied in all the runs.

### Proximity ligation assay (PLA)

Cells were fixed and permeabilised by treating 5 minutes with methanol (-20°C) and washing thrice with cold phosphate buffered saline. PLA was carried out according to the supplier’s protocol (Olink Bioscience). Primary antibodies used for the PLA reaction were anti-methyl Histone H3 Lysine 4 at a 1/50 dilution (Abcam ab8895) and anti HER2 at a 1/75 dilution (Abcam ab16901). Cells were observed with a Nikon Eclipse Ni-E microscope at magnification x630 and images were captured with Nikon’s NIS Elements Imaging software. DAPI and PLA fluorescence were captured at high resolution with automated 0.5μm z- sections for a total of 8 separate z-sections per observation field. All Z-sections were then stacked. Cell numeration and PLA labeling were carried out using Image J software. Cells and red PLA dots were counted using the ‘Analyze Particles’ function. For each condition, at least 200 cells were imaged and analyzed. A mean value of number of spots per nucleus was then calculated and normalised to the SKBR3 siNT sample. All statistical analyses were carried out by performing student’s T-Test.

### RNAseq

Total RNA was extracted from cells treated with vehicle or EGF (100ng/ml) for 3 hours. RNAseq libraries were prepared using Illumina’s TruSeq mRNA Stranded Sample prep kit. Five replicates were performed for each treatment.

### RNAseq data analysis

Single-end 40BP reads generated on the Illumina HiSeq were aligned to the human genome version GRCh37.64 using TopHat version 2.0.4 [35]. Read counts were then obtained using HTSeq-count version 0.5.3p9 (http://www-huber.embl.de/users/anders/HTSeq/doc/overview.html). Read counts were then normalised and tested for differential gene expression using the Bioconductor package *DESeq2* [36] version 1.8.1. Multiple testing correction was applied using the Benjamini-Hochberg method. Genes were selected as differentially expressed such that FDR <0.01.

### Integration of RNAseq and ChIP-seq data

Chip-seq/exo binding sites were annotated using genomic features of the Human Genome Assembly [GRCh37]. Genes whose body coordinates were in the proximity to chip-seq peak summits within region of +/- 10kbp were selected as potential target genes for regulation by HER2. P-values derived from DESeq2 analyses of the RNA-Seq data for all selected genes were–log10 transformed. These values were subsequently used for ranking and weighting of genes. GSEAPreranked analysis tool from Gene Set Enrichment Analysis (GSEA) software, version 2.2.3 (http://software.broadinstitute.org/gsea/index.jsp) has been used for establishing potential functional relation between Chip-seq/exo site location and gene expression. For hierarchical clustering, RNA-seq read counts were normalised using library size and standard deviation coefficients and then log2 transformed. Clustering functions and heatmap visualisation was performed using Matlab Bioinformatics toolbox framework (https://uk.mathworks.com/products/bioinfo.html).

### Western blot

Immunoblotting was performed using anti-ErbB2 (sc-284, Santa Cruz), anti-Tubulin (DM1a, Sigma-Aldrich), anti-Lamin B (ab20396, Abcam), anti-phospho-ERK1/2 (4370, Cell Signalling), anti-β-Actin (ab6276, Abcam).

In brief, whole cell lysates were extracted using the Pierce RIPA Buffer (Cat No 89900, Thermo Fisher Scientific) and quantified using the Bradford assay.

Chromatin fractionations were performed as follows: Cells pellets were resuspended in Buffer A [10mM Hepes (pH 7.9), 10mM KCl, 1.5mM MgCl2, 0.34M Sucrose, 10% glycerol] with 1mM DTT, protease inhibitors (PI), 0.1mM PMSF, 0.1% Triton X-100 and incubated on ice for 10 minutes. Nuclei were collected by centrifugation, 3,500rpm 5 minutes, 4°C. Supernatant was retained as cytosolic fraction. The pellet was washed once in Buffer A (plus 1mM DTT, PI, 0.1mM PMSF, no detergent) then resuspended in 200 μl of Buffer B [3mM EDTA, 0.2mM EGTA] with 1mM DTT, PI, 0.1mM PMSF and vortexed. Incubated on ice for 30 minutes with occasional vortexing, centrifuged at 4,000 rpm for 5 minutes at 4°C. The supernatant was retained as nucleoplasmic fraction. The pellet was wash pellet (x5) in 300 μl Buffer B (plus 1mM DTT, PI, 0.1mM PMSF). On last wash, sample was sonicated for 30 seconds then centrifuged at 5,000 rpm for 5 minutes at 4°C. The pellet was resuspended in 55 μl 1X DNase I Buffer (Ambion AM2222) plus 5 μl DNase and incubated at 37°C for 30–60 minutes. A needle and syringe were used to homogenise the sample if needed.

Samples were run on pre-cast NuPAGE^™^ 4–12% Bis-Tris Protein Gels (Thermo Fisher Scientific) until fully separated by molecular weight then transferred to a nitrocellulose membrane using iBlot^®^ Gel Transfer Device (Thermo Fisher Scientific) before incubation with blocking solution, following be primary and secondary antibodies. Proteins were detected using standard chemiluminescence.

### Data Deposition

The mass spectrometry proteomics data have been deposited to the ProteomeXchange Consortium via the PRIDE partner repository with the dataset identifier PXD003915. ChIPseq and RNAseq data have been deposited in the GEO database under the reference GSE79778.

## Supporting information

S1 Fig A) Western blot analysis using fractionated cell lysates from SKBR3 and BT474 cells, under asynchronous and EGF-treatment conditions. Cells were treated for 3 hours with 100ng/ml EGF. B) Comparison of HER2 ChIPexo and ChIPseq UCSC genome browser shots, illustrating the increased sensitivity of the ChIPexo method in detecting HER2 binding sites. C) Western blot analysis of cell lysates from SKBR3 cells after EGF treatment. Induction of pERK1/2 confirms the action of EGF in the treated cells. D) Venn diagram and UCSC genome browser shots illustrating the unique and common peaks in asynchronous and EGF-treated SKBR3 cells. E) CEAS analysis of the binding sites of HER2 in the SKBR3 cell line across various features of the genome.
(TIF)

S2 Fig A) Venn diagram and average intensity plots of HER2 ChIPexo binding sites in the BT474 cell line. The binding sites found in both the asynchronous and EGF-treated cells had the strongest average intensity. B) CEAS analysis of the binding sites of HER2 in the BT474 cell line across various features of the genome. C) Venn diagram illustrating the overlap between HER2 binding sites with H3K4me1 under EGF conditions in the BT474 cell line. D) Proximity ligation assay in the SKBR3 cell line utilising antibodies raised against HER2 and H3K4me1 illustrating an increase in the number of fluorescent foci with treatment of the EGF in comparison to control (PBS) treated cells. Anti-HER2 (mouse monoclonal, Abcam ab16901) and anti-H3K4me1 (rabbit polyclonal, Abcam ab8895) antibodies were used for PLA experiments. Histogram with quantification of fluorescent foci. *p-value < 0.05 (Student’s t-test). E) Coimmunoprecipitation in the SKBR3 cell line. EGFR and STAT3 were immunoprecipitated and western blot performed for HER2.
(TIF)

S1 Table HER2 RIME full data. Data from RIME experiments, from HER2 and IgG immunoprecipitations using nuclear and chromatin fractions from SKBR3 cell lines. In sample 602 & 603, EGF treated cells had been cultured in media containing heavy arginine and lysine, and vehicle treated cells had been cultured in media containing light arginine & lysine. In samples 628 & 629, the labels were reversed, i.e. the EGF treated cells had been cultured in media containing light arginine and lysine, and vehicle treated cells had been cultured in media containing heavy arginine & lysine.
(XLSX)

## References

[1] Curtis C, Shah SP, Chin SF, Turashvili G, Rueda OM, Dunning MJ, et al The genomic and transcriptomic architecture of 2,000 breast tumours reveals novel subgroups. Nature. 2012;486(7403):346–52. Epub 2012/04/24. 10.1038/nature10983 22522925 PMC3440846

[2] Slamon DJ, Clark GM, Wong SG, Levin WJ, Ullrich A, McGuire WL. Human breast cancer: correlation of relapse and survival with amplification of the HER-2/neu oncogene. Science. 1987;235(4785):177–82. Epub 1987/01/09. 10.1126/science.3798106 . 3798106

[3] Carter P, Presta L, Gorman CM, Ridgway JB, Henner D, Wong WL, et al Humanization of an anti-p185HER2 antibody for human cancer therapy. Proceedings of the National Academy of Sciences of the United States of America. 1992;89(10):4285–9. Epub 1992/05/15. 10.1073/pnas.89.10.4285 1350088 PMC49066

[4] Ryan Q, Ibrahim A, Cohen MH, Johnson J, Ko CW, Sridhara R, et al FDA drug approval summary: lapatinib in combination with capecitabine for previously treated metastatic breast cancer that overexpresses HER-2. The oncologist. 2008;13(10):1114–9. Epub 2008/10/14. 10.1634/theoncologist.2008-0816 . 18849320

[5] Krop IE, Kim SB, Martin AG, LoRusso PM, Ferrero JM, Badovinac-Crnjevic T, et al Trastuzumab emtansine versus treatment of physician’s choice in patients with previously treated HER2-positive metastatic breast cancer (TH3RESA): final overall survival results from a randomised open-label phase 3 trial. Lancet Oncol. 2017 10.1016/S1470-2045(17)30313-3 . 28526538

[6] Xie Y, Hung MC. Nuclear localization of p185neu tyrosine kinase and its association with transcriptional transactivation. Biochem Biophys Res Commun. 1994;203(3):1589–98. 10.1006/bbrc.1994.2368 . 7945309

[7] Giri DK, Ali-Seyed M, Li LY, Lee DF, Ling P, Bartholomeusz G, et al Endosomal transport of ErbB-2: mechanism for nuclear entry of the cell surface receptor. Molecular and cellular biology. 2005;25(24):11005–18. Epub 2005/11/30. 10.1128/MCB.25.24.11005-11018.2005 16314522 PMC1316946

[8] Beguelin W, Diaz Flaque MC, Proietti CJ, Cayrol F, Rivas MA, Tkach M, et al Progesterone receptor induces ErbB-2 nuclear translocation to promote breast cancer growth via a novel transcriptional effect: ErbB-2 function as a coactivator of Stat3. Molecular and cellular biology. 2010;30(23):5456–72. 10.1128/MCB.00012-10 20876300 PMC2976427

[9] Diaz Flaque MC, Galigniana NM, Beguelin W, Vicario R, Proietti CJ, Russo R, et al Progesterone receptor assembly of a transcriptional complex along with activator protein 1, signal transducer and activator of transcription 3 and ErbB-2 governs breast cancer growth and predicts response to endocrine therapy. Breast Cancer Res. 2013;15(6):R118 10.1186/bcr3587 24345432 PMC3978912

[10] Diaz Flaque MC, Vicario R, Proietti CJ, Izzo F, Schillaci R, Elizalde PV. Progestin drives breast cancer growth by inducing p21(CIP1) expression through the assembly of a transcriptional complex among Stat3, progesterone receptor and ErbB-2. Steroids. 2013;78(6):559–67. 10.1016/j.steroids.2012.11.003 . 23178160

[11] Wang SC, Lien HC, Xia W, Chen IF, Lo HW, Wang Z, et al Binding at and transactivation of the COX-2 promoter by nuclear tyrosine kinase receptor ErbB-2. Cancer cell. 2004;6(3):251–61. Epub 2004/09/24. 10.1016/j.ccr.2004.07.012 . 15380516

[12] Venturutti L, Romero LV, Urtreger AJ, Chervo MF, Cordo Russo RI, Mercogliano MF, et al Stat3 regulates ErbB-2 expression and co-opts ErbB-2 nuclear function to induce miR-21 expression, PDCD4 downregulation and breast cancer metastasis. Oncogene. 2016;35(17):2208–22. 10.1038/onc.2015.281 . 26212010

[13] Li LY, Chen H, Hsieh YH, Wang YN, Chu HJ, Chen YH, et al Nuclear ErbB2 enhances translation and cell growth by activating transcription of ribosomal RNA genes. Cancer Res. 2011;71(12):4269–79. 10.1158/0008-5472.CAN-10-3504 21555369 PMC3117049

[14] Li X, Kuang J, Shen Y, Majer MM, Nelson CC, Parsawar K, et al The atypical histone macroH2A1.2 interacts with HER-2 protein in cancer cells. The Journal of biological chemistry. 2012;287(27):23171–83. 10.1074/jbc.M112.379412 22589551 PMC3391143

[15] Dillon MF, Stafford AT, Kelly G, Redmond AM, McIlroy M, Crotty TB, et al Cyclooxygenase-2 predicts adverse effects of tamoxifen: a possible mechanism of role for nuclear HER2 in breast cancer patients. Endocrine-related cancer. 2008;15(3):745–53. Epub 2008/05/13. 10.1677/ERC-08-0009 . 18469157

[16] Schillaci R, Guzman P, Cayrol F, Beguelin W, Diaz Flaque MC, Proietti CJ, et al Clinical relevance of ErbB-2/HER2 nuclear expression in breast cancer. BMC cancer. 2012;12:74 Epub 2012/02/24. 10.1186/1471-2407-12-74 22356700 PMC3342900

[17] Cordo Russo RI, Beguelin W, Diaz Flaque MC, Proietti CJ, Venturutti L, Galigniana N, et al Targeting ErbB-2 nuclear localization and function inhibits breast cancer growth and overcomes trastuzumab resistance. Oncogene. 2015;34(26):3413–28. Epub 2014/09/02. 10.1038/onc.2014.272 . 25174405

[18] Serandour AA, Brown GD, Cohen JD, Carroll JS. Development of an Illumina-based ChIP-exonuclease method provides insight into FoxA1-DNA binding properties. Genome biology. 2013;14(12):R147 Epub 2014/01/01. 10.1186/gb-2013-14-12-r147 . 24373287 PMC4053927

[19] Zhang Y, Liu T, Meyer CA, Eeckhoute J, Johnson DS, Bernstein BE, et al Model-based analysis of ChIP-Seq (MACS). Genome biology. 2008;9(9):R137 Epub 2008/09/19. 10.1186/gb-2008-9-9-r137 18798982 PMC2592715

[20] Heintzman ND, Stuart RK, Hon G, Fu Y, Ching CW, Hawkins RD, et al Distinct and predictive chromatin signatures of transcriptional promoters and enhancers in the human genome. Nat Genet. 2007;39(3):311–8. 10.1038/ng1966 . 17277777

[21] Poulard C, Rambaud J, Le Romancer M, Corbo L. Proximity ligation assay to detect and localize the interactions of ERalpha with PI3-K and Src in breast cancer cells and tumor samples. Methods Mol Biol. 2014;1204:135–43. 10.1007/978-1-4939-1346-6_12 . 25182767

[22] Mootha VK, Lindgren CM, Eriksson KF, Subramanian A, Sihag S, Lehar J, et al PGC-1alpha-responsive genes involved in oxidative phosphorylation are coordinately downregulated in human diabetes. Nature genetics. 2003;34(3):267–73. Epub 2003/06/17. 10.1038/ng1180 . 12808457

[23] Subramanian A, Tamayo P, Mootha VK, Mukherjee S, Ebert BL, Gillette MA, et al Gene set enrichment analysis: a knowledge-based approach for interpreting genome-wide expression profiles. Proc Natl Acad Sci U S A. 2005;102(43):15545–50. 10.1073/pnas.0506580102 16199517 PMC1239896

[24] Mohammed H, D’Santos C, Serandour AA, Ali HR, Brown GD, Atkins A, et al Endogenous purification reveals GREB1 as a key estrogen receptor regulatory factor. Cell Rep. 2013;3(2):342–9. 10.1016/j.celrep.2013.01.010 . 23403292 PMC7116645

[25] Lin SY, Makino K, Xia W, Matin A, Wen Y, Kwong KY, et al Nuclear localization of EGF receptor and its potential new role as a transcription factor. Nature cell biology. 2001;3(9):802–8. Epub 2001/09/05. 10.1038/ncb0901-802 . 11533659

[26] Andrique L, Fauvin D, El Maassarani M, Colasson H, Vannier B, Seite P. ErbB3(80 kDa), a nuclear variant of the ErbB3 receptor, binds to the Cyclin D1 promoter to activate cell proliferation but is negatively controlled by p14ARF. Cellular signalling. 2012;24(5):1074–85. Epub 2012/01/21. 10.1016/j.cellsig.2012.01.002 . 22261253

[27] Wang YN, Lee HH, Lee HJ, Du Y, Yamaguchi H, Hung MC. Membrane-bound trafficking regulates nuclear transport of integral epidermal growth factor receptor (EGFR) and ErbB-2. The Journal of biological chemistry. 2012;287(20):16869–79. Epub 2012/03/28. 10.1074/jbc.M111.314799 22451678 PMC3351284

[28] Dawson MA, Bannister AJ, Gottgens B, Foster SD, Bartke T, Green AR, et al JAK2 phosphorylates histone H3Y41 and excludes HP1alpha from chromatin. Nature. 2009;461(7265):819–22. 10.1038/nature08448 19783980 PMC3785147

[29] Tiwari VK, Stadler MB, Wirbelauer C, Paro R, Schubeler D, Beisel C. A chromatin-modifying function of JNK during stem cell differentiation. Nat Genet. 2012;44(1):94–100. 10.1038/ng.1036 . 22179133

[30] Schmidt D, Wilson MD, Spyrou C, Brown GD, Hadfield J, Odom DT. ChIP-seq: using high-throughput sequencing to discover protein-DNA interactions. Methods. 2009;48(3):240–8. Epub 2009/03/12. 10.1016/j.ymeth.2009.03.001 . 19275939 PMC4052679

[31] Langmead B, Salzberg SL. Fast gapped-read alignment with Bowtie 2. Nat Methods. 2012;9(4):357–9. 10.1038/nmeth.1923 22388286 PMC3322381

[32] Zang C, Schones DE, Zeng C, Cui K, Zhao K, Peng W. A clustering approach for identification of enriched domains from histone modification ChIP-Seq data. Bioinformatics. 2009;25(15):1952–8. 10.1093/bioinformatics/btp340 19505939 PMC2732366

[33] Consortium EP. An integrated encyclopedia of DNA elements in the human genome. Nature. 2012;489(7414):57–74. 10.1038/nature11247 22955616 PMC3439153

[34] Bailey TL, Boden M, Buske FA, Frith M, Grant CE, Clementi L, et al MEME SUITE: tools for motif discovery and searching. Nucleic Acids Res. 2009;37(Web Server issue):W202–8. 10.1093/nar/gkp335 19458158 PMC2703892

[35] Trapnell C, Pachter L, Salzberg SL. TopHat: discovering splice junctions with RNA-Seq. Bioinformatics. 2009;25(9):1105–11. 10.1093/bioinformatics/btp120 19289445 PMC2672628

[36] Love MI, Huber W, Anders S. Moderated estimation of fold change and dispersion for RNA-seq data with DESeq2. Genome biology. 2014;15(12):550 10.1186/s13059-014-0550-8 25516281 PMC4302049

