# Supplementary figures and images for: Analysis of HER2 genomic binding in breast cancer cells identifies a global role in direct gene regulation
Source: PLoS One. 2019 Nov 20;14(11):e0225180. doi: 10.1371/journal.pone.0225180 (PMC6867699; doi:10.1371/journal.pone.0225180)

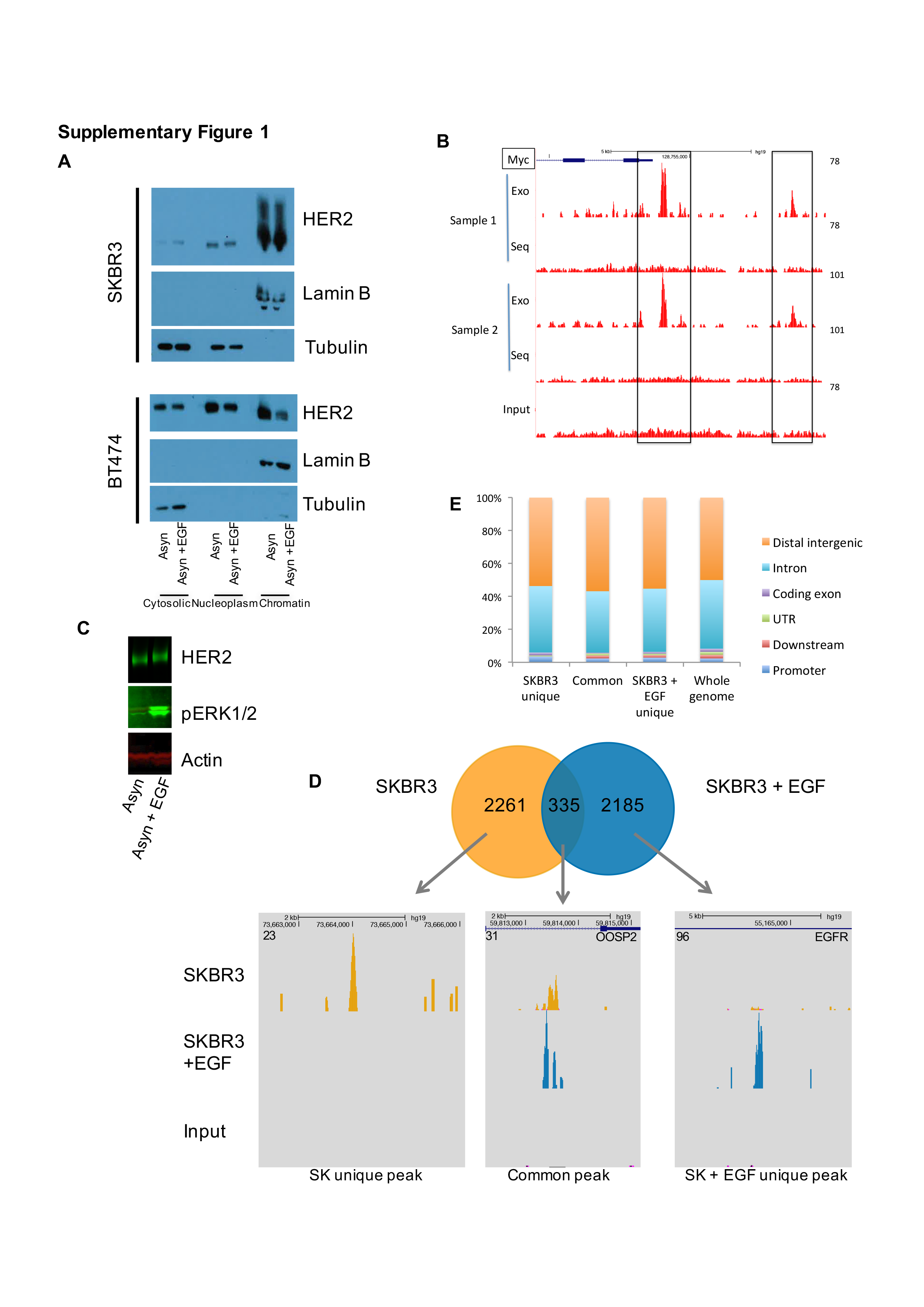

Supplement: S1 Fig — A) Western blot analysis using fractionated cell lysates from SKBR3 and BT474 cells, under asynchronous and EGF-treatment conditions. Cells were treated for 3 hours with 100ng/ml EGF. B) Comparison of HER2 ChIPexo and ChIPseq UCSC genome browser shots, illustrating the increased sensitivity of the ChIPexo method in detecting HER2 binding sites. C) Western blot analysis of cell lysates from SKBR3 cells after EGF treatment. Induction of pERK1/2 confirms the action of EGF in the treated cells. D) Venn diagram and UCSC genome browser shots illustrating the unique and common peaks in asynchronous and EGF-treated SKBR3 cells. E) CEAS analysis of the binding sites of HER2 in the SKBR3 cell line across various features of the genome. (TIF) [file pone.0225180.s001.tif]

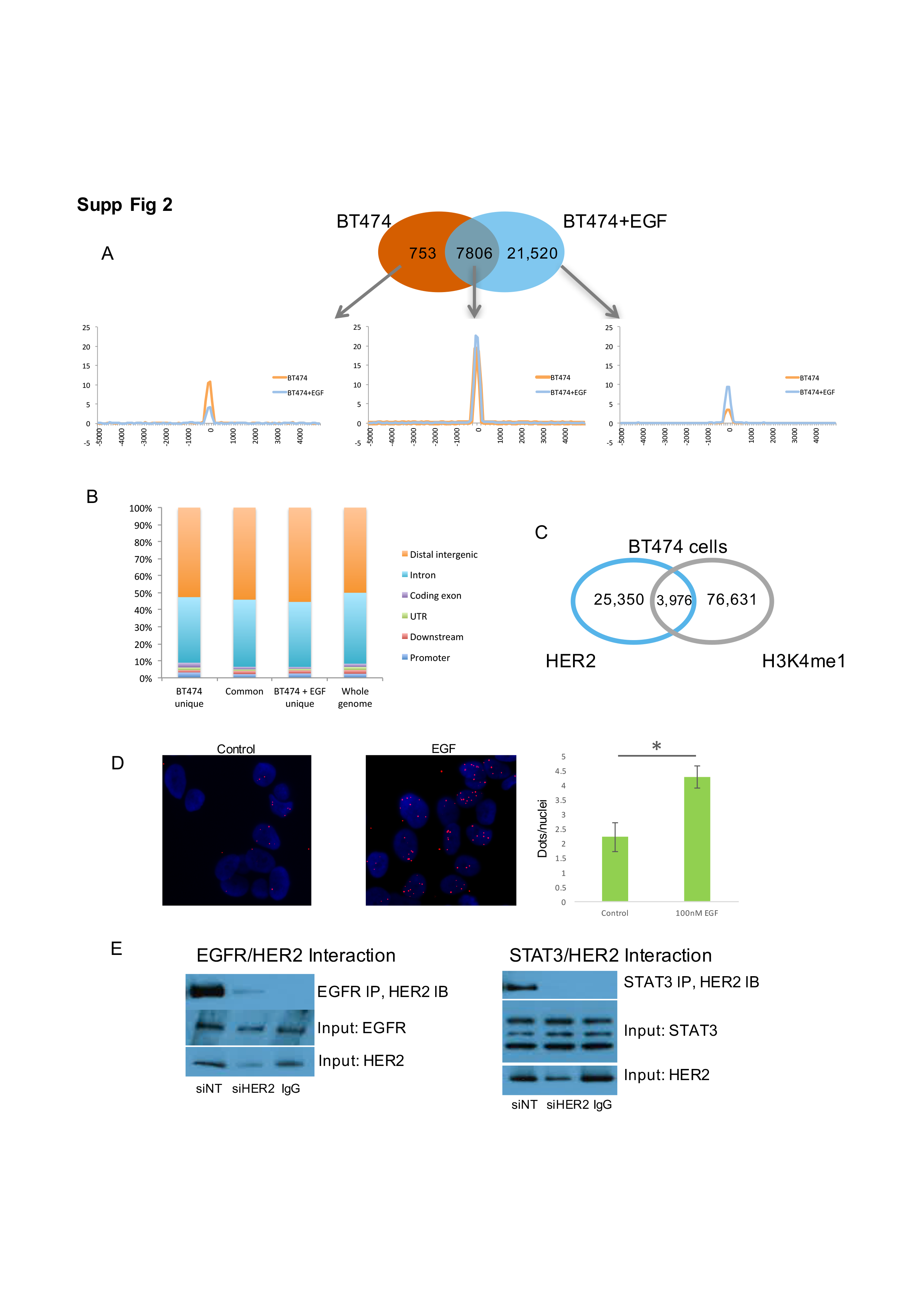

Supplement: S2 Fig — A) Venn diagram and average intensity plots of HER2 ChIPexo binding sites in the BT474 cell line. The binding sites found in both the asynchronous and EGF-treated cells had the strongest average intensity. B) CEAS analysis of the binding sites of HER2 in the BT474 cell line across various features of the genome. C) Venn diagram illustrating the overlap between HER2 binding sites with H3K4me1 under EGF conditions in the BT474 cell line. D) Proximity ligation assay in the SKBR3 cell line utilising antibodies raised against HER2 and H3K4me1 illustrating an increase in the number of fluorescent foci with treatment of the EGF in comparison to control (PBS) treated cells. Anti-HER2 (mouse monoclonal, Abcam ab16901) and anti-H3K4me1 (rabbit polyclonal, Abcam ab8895) antibodies were used for PLA experiments. Histogram with quantification of fluorescent foci. *p-value < 0.05 (Student’s t-test). E) Coimmunoprecipitation in the SKBR3 cell line. EGFR and STAT3 were immunoprecipitated and western blot performed for HER2. (TIF) [file pone.0225180.s002.tif]
